# A single‐needle puncture technique to perform sciatic and adductor canal blocks for below‐knee surgery

**DOI:** 10.1002/anr3.12211

**Published:** 2023-02-20

**Authors:** J. W. L. Lim, Y. L. Cheng

**Affiliations:** ^1^ Department of Anaesthesiology Ng Teng Fong General Hospital Singapore

**Keywords:** anaesthesia, conduction, lower extremity, nerve block, sciatic nerve

Performing sciatic nerve and adductor canal blocks with a single‐needle puncture in the supine position has the potential to increase patient comfort and reduce procedural time. We performed this technique to facilitate urgent surgery for a patient at high risk of complications from general anaesthesia, in whom central neuraxial blockade was contraindicated.

A 52‐year‐old female with a background medical history of diabetes mellitus and ischaemic cardiomyopathy (ejection fraction 40%) was scheduled for urgent right ankle wound debridement and application of antibiotic beads and negative pressure dressings. She was initially admitted to hospital for management of necrotising fasciitis of the right leg and had undergone several soft tissue debridement procedures during her admission. Closure of the affected area had been attempted with a free flap procedure, but this had failed due to tissue breakdown. The patient was receiving prophylactic doses of subcutaneous low molecular weight heparin (LMWH) due to prolonged bed rest.

On the night proceeding surgery, the patient's clinical condition deteriorated. Clinical examination revealed bilateral chest crepitations and pedal oedema. A chest radiograph was consistent with pulmonary oedema. The patient was treated with furosemide 40 mg intravenously. The surgical team wished to proceed urgently due to the high risk to the patient of developing osteomyelitis should the debridement be delayed. Following multidisciplinary discussion, we agreed to facilitate early surgery, within 12 h of the patient's last dose of LWMH.

As the patient displayed signs of worsening cardiac failure, proceeding with general anaesthesia was judged to confer a high risk of peri‐operative cardiovascular complications. Spinal anaesthesia was contraindicated due to the administration of prophylactic LMWH 6 h pre‐operatively [1].

Given the urgency of this procedure and the patient's precarious clinical status, superficial peripheral nerve block was the anaesthetic technique of choice due to the relatively lower risks associated with bleeding compared with central neuraxial blockade. A superficial nerve block has a lower risk of clinically significant consequences of bleeding and is performed at a location where management of bleeding complication is relatively straightforward [1]. The patient consented to this technique.

**Figure 1:**
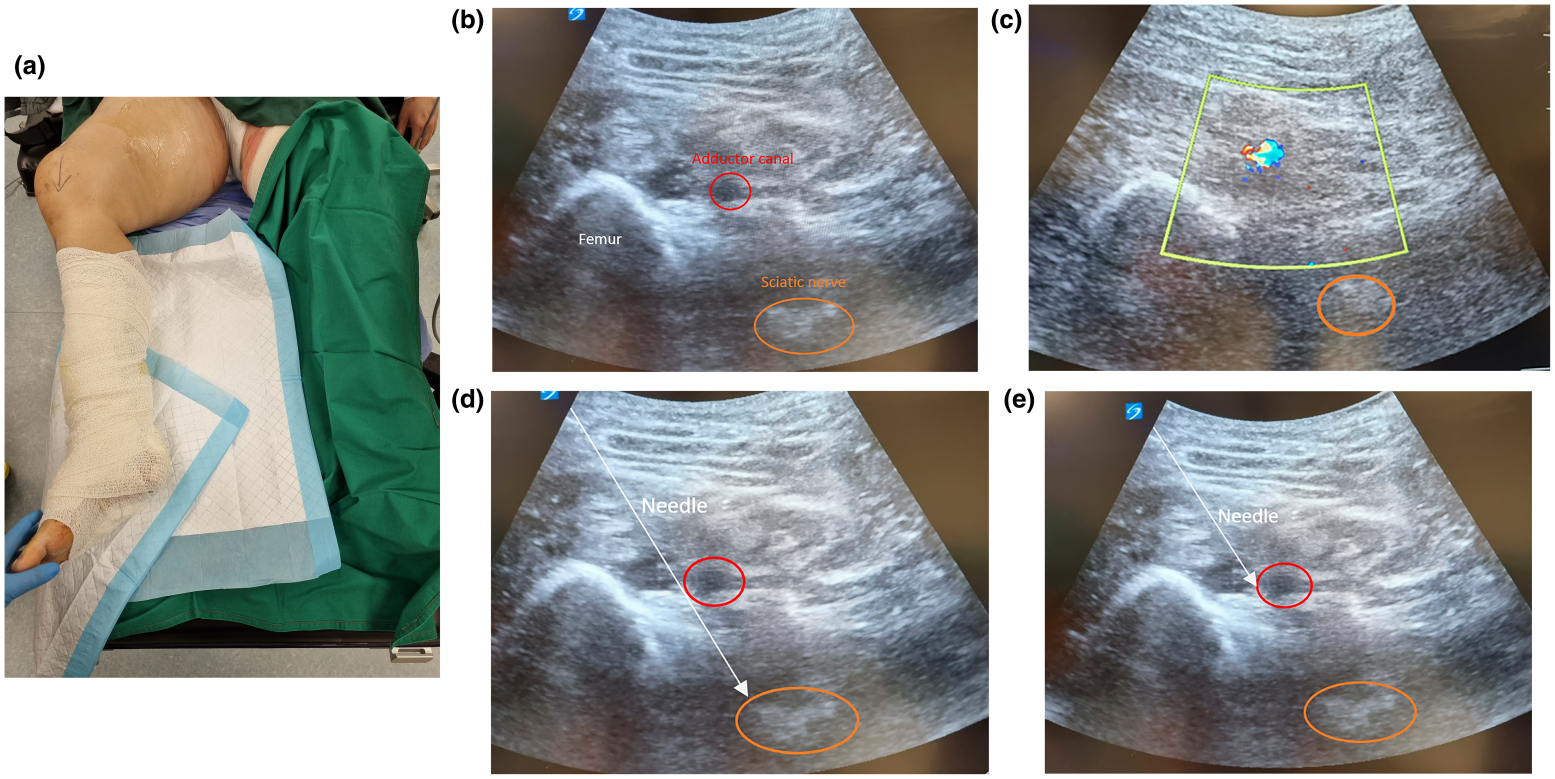
(a) Position of patient's right lower limb for sciatic and adductor canal block; (b) ultrasound imaging of both sciatic nerve and adductor canal; (c) colour Doppler of superficial femoral artery; (d) local anaesthetic deposited around the sciatic nerve; (e) local anaesthetic deposited in the adductor canal.

Pre‐operatively, examination of the patient's right leg revealed no sensory or motor deficit. Standard anaesthesia monitoring was established, and the patient remained in the supine position throughout block administration. Her leg was positioned with the hip externally rotated and knee flexed (Fig. 1a).

A target‐controlled infusion of propofol (plasma target concentration 1.5 μg.ml^−1^) was commenced to provide sedation, titrated to maintain purposeful response to verbal instructions. A curvilinear ultrasound probe (Sonosite X‐Porte, Fujifulm Sonosite, Inc., Bothell, WA, USA) was used to guide nerve blockade. The probe was placed on the mid medial right thigh to obtain the ultrasound image of both the sciatic nerve and adductor canal (Fig. 1b,c). Three millilitres of 1% lignocaine were injected subcutaneously prior to the insertion of a 100 mm echogenic needle (SonoPlex, Pajunk, Geisingen, Germany) under real‐time ultrasound imaging using an in‐plane technique. After confirming the position of the needle tip on both ultrasound images, 20 ml of 0.4% ropivacaine and 15 ml of 0.8% lignocaine was deposited around the sciatic nerve (Fig. 1d), and into the adductor canal (Fig. 1e).

The surgical procedure was performed uneventfully. The patient did not require any further analgesia intra‐ or postoperatively. She regained full sensory and motor function of her right lower limb approximately 10 h after completion of both blocks, and there were no block‐related complications. When reviewed postoperatively, the patient reported that she was highly satisfied with the anaesthesia performed. Pre‐operatively she had been concerned that changing position to allow the nerve blocks to be performed might have been uncomfortable. Additionally, having one needle puncture was preferable to multiple punctures due to a phobia of needles.

This approach is useful for patients who have problems changing their position due to pain or physical limitations. Our experience is that this is an effective and efficient technique, both for analgesia and as the sole anaesthetic technique for below‐knee surgery. It reduces the time needed, number of needle punctures and staffing requirements to achieve sensory blockade for surgery below the knee.
